# Pachychoroid disease: review and update

**DOI:** 10.1038/s41433-024-03253-4

**Published:** 2024-08-03

**Authors:** Chui Ming Gemmy Cheung, Kunal K. Dansingani, Hideki Koizumi, Timothy Y. Y. Lai, Sobha Sivaprasad, Camiel J. F. Boon, Elon H. C. Van Dijk, Jay Chhablani, Won Ki Lee, K. Bailey Freund

**Affiliations:** 1 Singapore National Eye Centre, Singapore, Singapore; 2 Singapore Eye Research Institute, Singapore, Singapore; 3 Duke-NUS Medical School, Singapore, Singapore; 4 Department of Ophthalmology, University of Pittsburgh Medical Center, Pittsburgh, PA USA; 5 Department of Ophthalmology, Graduate School of Medicine, University of the Ryukyus, Okinawa, Japan; 6 Department of Ophthalmology and Visual Sciences, The Chinese University of Hong Kong, Hong Kong, China; 7 Moorfields Clinical Research Facility, NIHR Biomedical Research Centre, Moorfields Eye Hospital NHS Foundation Trust, London, UK; 8 Amsterdam University Medical Centers, Amsterdam, the Netherlands; 9 Department of Ophthalmology, Leiden University Medical Center, Leiden, the Netherlands; 10 Nune Eye Hospital, Seoul, South Korea; 11 Vitreous Retina Macula Consultants of New York, New York, NY USA; 12 Department of Ophthalmology, NYU Grossman School of Medicine, New York, NY USA

**Keywords:** Eye diseases, Pathogenesis

## Abstract

The pachychoroid disease spectrum is a phenotype characterized by alterations in choroidal vasculature which result in outer retinal and choriocapillaris damage and visual loss. The presence of pachyvessels is one of the key features of the pachychoroid phenotype. Recent imaging studies suggest that pachyvessels may form because of choroidal venous congestion in one or more quadrants. The formation of intervortex anastomosis may function as a compensatory mechanism to dissipate the increased venous pressure, while outflow obstruction has been hypothesized to occur at the site of the vortex vein exiting the sclera. This review aims to summarize recent imaging findings and discuss evolution in the understanding of pathogenesis of the pachychoroid disease spectrum. We have summarized notable treatment trials in central serous chorioretinopathy and polypoidal choroidal vasculopathy and included an update of the current diagnostic and management strategies of the entities that are part of the pachychoroid disease spectrum.

## Abstract (zh)

肥厚型脉络膜疾病谱是一种以脉络膜血管改变为特征的一组疾病, 可导致视网膜和脉络膜毛细血管损伤和视力丧失。肥厚性血管是肥厚型脉络膜表型的主要特征之一。最新影像学研究表明, 肥厚血管的成因可能是一个或多个象限的脉络膜静脉充血。涡静脉血管吻合的形成可能是一种代偿机制, 以抵消升高的静脉压, 而流出阻塞可能发生在涡旋静脉出巩膜的位置。本综述旨在总结最新的影像学表现并论述肥厚型脉络膜疾病谱发病机制的研究进展。我们总结了中心性浆液性视网膜脉络膜病变和息肉样脉络膜病变的值得关注的一些临床试验, 并对肥厚型脉络膜疾病谱的分型以及当前诊断和管理的策略进行了更新。

## Introduction

The term “pachychoroid” was first introduced to describe the thickened choroid seen in eyes with central serous chorioretinopathy (CSC) and in eyes without subretinal fluid (SRF) showing retinal pigment epithelium (RPE) alterations appearing to be mechanistically related to a choroidal abnormality like that producing CSC [1]. Since not all eyes with thick choroid show fundus abnormalities, the term “pachychoroid disease” was introduced to indicate a more refined choroidal phenotype associated with a spectrum of retinal diseases which includes CSC, pachychoroid pigment epitheliopathy (PPE), pachychoroid neovasculopathy (PNV), peripapillary pachychoroid syndrome (PPS), and polypoidal choroidal vasculopathy (PCV). Since the publication of our previous review in 2018 [2], important new observations have advanced our understanding of the pathogenesis of pachychoroid disease. This review will focus on peer-reviewed published manuscripts after 2018 based on a PubMed search relevant to the pachychoroid disease. It will also discuss limitations of the current nomenclature which has led to some confusion regarding the definition of this phenotype.

Advances in imaging techniques have led to the observation of choroidal venous abnormalities which support a growing consensus that choroidal venous congestion is a primary driver of pachychoroid diseases. Abnormalities in the larger choroidal veins have taken center-stage in the pachychoroid phenotype, and their presence has superseded choroidal thickness measurement in defining this phenotype. Importantly, there are several hypothesized explanations as to how alterations in large choroidal veins may lead to choriocapillaris and RPE damage manifesting in the form of pachychoroid diseases. Concurrently, there have been advances in detecting, monitoring, and treating the different diseases that are part of the pachychoroid disease spectrum.

## Limitation of choroidal thickness

Although initial descriptions of pachychoroid disease reported an association with increased choroidal thickness (CT) [1, 3], more recent studies suggest CT is not the most salient disease-defining feature. The range of CT reported in previous studies for healthy eyes is broad (191–350 μm) [4], and may be influenced by factors including age, axial length, blood pressure, and diurnal variations. In comparison, CT in the range of 345–505 μm for CSC [5, 6] and 223–590 μm for PCV [7] have been reported. A wide range of CT has been noted in both conditions. In PCV, even a bimodal distribution of CT has been described, with peaks at 170 and 390 µm [8]. Since the CT distributions in healthy eyes and diseased eyes overlap considerably and a wide range is observed generally, there is currently no universally accepted threshold above which CT is considered as abnormal. Furthermore, in eyes with thick choroid, choroidal vascular markings can be indistinct, and attenuation of OCT signal in the outer choroid and choroidal–scleral interface may also lead to inaccuracies in thickness measurements. A further limitation of choroidal thickness measurements is the inability to differentiate between vascular and stromal components of the choroid.

Beyond studying sub-foveal CT, regional CT variation may provide additional insight into the choroidal pathology related to pachychoroid disease. CT mapping in chronic CSC and PPS have demonstrated a shift in the site of maximum thickness closer to the site of RPE leakage, which may be extrafoveal. This is often described as focal choroidal thickening [9, 10].

## New observations related to choroidal venous alterations

**Fig. 1:**
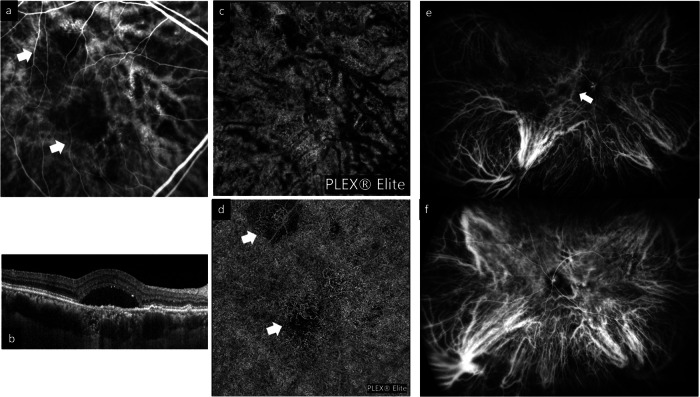
Examples of imaging findings related to choroidal alterations in the pachychoroid disease spectrum. Figure 1a shows the presence of pachyvessels which appear as prominent choroidal veins with large caliber crossing the horizontal watershed on indocyanine green angiography (ICGA). Areas of reduced filling are indicated by white arrows (**a**). This eye is also imaged with OCT (**b**) and en face 12 × 12 mm OCTA (**c**, **d**). Presence of subretinal fluid and pachyvessels within a thickened choroid can be observed in (**b**). **c** The pachyvessels can be observed with en face OCT using a choroidal slab and closely correspond to those seen on ICGA in (**a**). **d** Several areas of choriocapillaris flow impairment can be observed with en face OCTA using the choriocapillaris slab (white arrows). These areas correspond closely to the areas of reduced filling on ICGA in (**a**). **e**, **f** Ultra-widefield (UWF) ICGA can be used to evaluate the arrangement of vortex veins. The example in (**e**) shows a dilated and congested vortex vein in the inferotemporal quadrant. Anastomosis can be observed between the inferotemporal and superotemporal quadrants (white arrow). The example in (**f**) shows widespread dilated and congested vortex veins in all four quadrants. Multiple anastomosis can be observed around the optic disc.

In our previous review, we highlighted the following characteristics shared among eyes with pachychoroid disease: 1) increased CT; 2) presence of pachyvessels (discussed in “New observations related to choroidal venous alterations” a) ; 3) inner choroid attenuation; and 4) choroidal vascular hyperpermeability [2]. While the emphasis on CT has since then declined, recent findings, including ultra-widefield (UWF) imaging, have provided further understanding related to the nature of choroidal venous congestion [11–13]. Inner choroidal ischemia continues to be regarded as an important sequela of pachychoroid disease which may result in neovascular complications and macular atrophy [14, 15]. (Fig. 1) The role of the sclera in influencing pachychoroid disease has also gained attention, with cumulative evidence suggesting increased thickness of both the anterior and posterior sclera to be risk factors for pachychoroid disease [16]. Thickened sclera could impose resistance to venous outflow to the vortex veins, and to the transscleral passage of proteins and fluid.

## Choroidal venous alterations

The presence of pachyvessels has been considered a hallmark of the pachychoroid disease phenotype. Warrow and Freund first described dilated submacular choroidal vessels in eyes with PPE [1]. Subsequently, en face OCT studies observed dilated outer choroidal vessels correlated to disease foci in PPE [9]. The affected vessels often appeared to have originated in Haller’s layer and exhibited increased caliber at the origin of the vein within the posterior pole. The term “pachyvessel” was introduced to describe these vessels, but the mechanisms accounting for their occurrence was uncertain at the time. Recently described findings suggest that pachyvessels may result from remodeling of Haller’s layer influenced by altered pressure gradients on opposite sides of choroidal watershed zones. Furthermore, these changes often extend outside the macula to the drainage areas of the vortex vein system [13].

### Choroidal drainage arrangement in health

Data regarding the normal distribution of venous drainage to each vortex system are limited. Hayreh first illustrated the segmental organization of the choroidal venous system into four quadrants, with an absence of anastomoses between separate vortex vein systems in healthy eyes [17], but later studies described preferential drainage through the superotemporal or inferotemporal route in over half of healthy volunteers [18]. Inter-individual variation in choroidal venous arrangement exists, with some studies reporting as many as eight vortex ampullae in healthy eyes [11].

Also, the number of vortex ampullae observed with fundus imaging is often higher than the number of vortex veins seen exiting the sclera as described in histological studies. This discrepancy suggests that some vortex ampullae may merge intra-sclerally before exiting the globe.

### Choroidal remodeling and macular anastomosis

A horizontal watershed zone within the macular region is frequently observed due to limited communication between the superior and inferior vortex veins in this area [19]. Hence, the finding of choroidal vessels with increased caliber crossing the macula has led researchers to investigate venous remodeling as a mechanism related to pachychoroid disease [20, 21]. Matsumoto et al. observed dilated choroidal vessels violating the horizontal watershed zone in >90% of eyes with pachychoroid diseases, versus in only 20% of control eyes [22]. The authors hypothesized that venous remodeling through formation of anastomoses between the superior and inferior vortex veins may improve venous drainage in eyes with chronic venous congestion.

Concurrently, studies using dynamic indocyanine green angiography (ICGA) observed delayed and pulsatile filling of the pachyvessels [23]. In some eyes, a retrograde pattern with back-and-forth oscillation was observed. The authors proposed that these observations may result from decreased perfusion pressure within affected vessel segments, possibly resulting from outflow abnormalities of the choroidal venous system [24].

### Vortex vein anatomy and pachychoroid disease

Pang et al. [12, 25]. described engorgement of the vortex vein ampullae in eyes with CSC using UWF ICGA, and hypothesized that outflow congestion may contribute to the pathogenesis of the pachychoroid disease spectrum [25]. Adoption of UWF imaging has allowed researchers to examine the anatomy of the vortex vein systems from the posterior pole to the ampullae. While prior imaging studies had described pachyvessels violating the horizontal physiological watershed zone in the macula, UWF studies revealed some pachyvessels communicating across quadrants in the periphery, providing further support for the hypothesis that pachyvessels result from venous remodeling to provide intervortex venous anastomosis to alleviate pathological choroidal congestion [20, 26, 27] (see “Evolution in the understanding of pathogenesis”). [24] However, the variation in vortex veins arrangement in healthy eyes suggest that it is possible that the presence of anastomosis may not always indicate underlying pathology [28, 29].

In addition to anastomoses, asymmetry in choroidal drainage distribution and focal constrictions/dilatations have been described in >90% of eyes with pachychoroid diseases [13, 24, 30]. This imbalance in choroidal venous drainage may predispose certain individuals with relatively hypoplastic vortex veins in 1 or more quadrants to develop congestion within the remaining vortex vein systems. Whether venous anastomosis is always pathological remains unclear, as anastomoses have been observed in 38–44.4% of normal eyes and have not been found to correlate with age [28, 30]. The significance of these findings in normal eyes remains uncertain, although some researchers proposed that eyes with intervortex venous anastomoses without disease manifestation may have subclinical forms of choroidal venous congestion [29]. Macular anastomoses have also been detected in 58% of eyes with typical neovascular age-related macular degeneration (AMD) in which the mean subfoveal CT was only 167 µm [31]. In addition to increase in vessel caliber, variation in the caliber of choroidal vessels leading to a sausage or bulbous appearance has been observed in eyes with CSC and PCV [31, 32]. The mechanism leading to these changes has not been fully elucidated, but current hypotheses include changes in venous pressure and vessel wall degeneration [31, 32].

Despite much progress, certain aspects of the nature of pachyvessels remain uncharacterized, such as whether they all function as intervortex venous anastomoses [24]. Even with UWF ICGA, en face SS-OCT, and en face SS-OCTA, the complexity of the choroidal vascular anatomy may preclude confirmation of true vascular connections across choroidal watershed zones. Furthermore, information on flow dynamics, such as direction, velocity, and oxygen tension within the choroidal vessels, cannot be assessed readily with current conventional imaging modalities. A further point of uncertainty regarding pachyvessels relates to whether they are all venous. Even though intervortex anastomoses resemble veins more than arteries, the fact that they appear to carry blood from 1 ampulla to another probably disqualifies them from being described as either veins or arteries. They do not seem to drain choroidal lobules but rather run from vein to vein, acting as bypass or overflow channels. Therefore, the theory regarding the pachychoroid phenotype is expected to continue to evolve as new observations become available.

## Choriocapillaris

Choriocapillaris impairment has been implicated in pachychoroid disease and is believed to contribute to visual loss in many eyes. Imaging the choriocapillaris, however remains technically challenging.

### ICGA and structural OCT evaluation of the choriocapillaris

ICGA shows regional choroidal hyperpermeability in virtually all CSC eyes with preserved choriocapillaris [24]. In eyes with focal atrophy of the choriocapillaris, hyperpermeability may be lost and the ICGA images show, instead, well-delineated Haller’s layer vessels on a hypofluorescent background. Leakage in the mid- to late- phase of ICGA has also been reported in eyes with pachychoroid disease, as well as fellow eyes of patients with unilateral subretinal fluid leakage in the context of CSC, and may be one of the most typical findings in this disease spectrum [24, 33, 34]. Intriguingly, CSC is rarely associated with myopia. Myopic patients with CSC generally have a normal-to-thin choroid, but choroidal hyperfluorescence on ICGA typical of CSC [35] is helpful to support the diagnosis. While a hyperpermeable choroid may result from a variety of conditions, such as inflammation and protein-losing disorders, the typical ICGA leakage observed in pachychoroid disease exhibits close topographic correlation with the pachyvessels and frequently co-localizes with foci of disease activity [24]. In the early phase of ICGA, these areas exhibit hypofluorescence or delayed filling, suggesting a degree of choriocapillaris flow disturbance [36]. The field of view of early ICGA imaging systems was previously limited to <50°, restricting analyses to the posterior pole. UWF imaging subsequently showed the manifestations of CSC to be more extensive [9, 14]. UWF fundus autofluorescence showed that RPE changes and gravitating tracks could occur as far as the equator and that choroidal venous dilatation could often involve the entire length of affected veins all the way to their respective ampullae. These findings form the foundation for the current mechanistic model for CSC. Using sequential frame subtraction of dynamic ICGA filling sequence, the filling of choriocapillaris units was elucidated in non-human primates [37]. The observations suggest that the pressure gradient between the arteriole inflow and venous drainage determines the filling characteristics of the functional choroidal lobule. It follows that venous outflow obstruction can lead to choriocapillaris ischemia through a reduction in perfusion pressure in the choroidal lobules. This ischemic environment may predispose towards the development of neovascular complications that are part of the pachychoroid disease spectrum, such as PNV and PCV.

Alterations in choroidal morphology on OCT cross-sections have been summarized in our previous review as enlarged choroidal veins appearing to originate from Haller’s layer, relative preservation of Sattler’s layer, and thinning or loss of the choriocapillaris in the overlying region [2]. These morphological changes within the choroid are also more prominent at the sites of RPE leakage and neurosensory detachment and, additionally, at sites of RPE change and neovascularization in PPE and PNV, respectively. Focal choroidal thickening with an increased Haller-to-choriocapillaris thickness ratio is also seen in PPE, PNV, and its aneurysmal/polypoidal variant, PCV.^8^

### OCTA evaluation of the choriocapillaris

Reduction in choriocapillaris flow has been documented in eyes with pachychoroid disease in several en face OCTA studies [14, 15, 38]. Although the individual vessels of the choriocapillaris are too small to resolve, uniformity of the choriocapillaris texture can be appreciated subjectively and by quantification of foci where this texture is lost. Foci of absent OCTA signal (“flow signal deficits” or “signal deficits”) occur in healthy eyes and Spaide has shown that the relationship between their size and number follows a power law distribution [39]. The logarithmic slope of this relationship has been shown to be altered in eyes with pachychoroid disease, but not in eyes with increased CT without disease [15].

Technically, the pachychoroid phenotype is also more challenging to study because tissue changes in the RPE and retina can confound segmentation of the choriocapillaris and/or mask choriocapillaris visualization by OCT, introducing artifacts into the analysis. Another technical limitation of OCTA is the inability to segment the transition from choriocapillaris to Sattler’s layer, as it is not possible to resolve the vessels of the choriocapillaris itself. Studying Sattler’s layer in isolation is therefore difficult and the literature on this is sparse. Haller’s layer vessels are poorly visualized by OCTA. It has been suggested that their flow velocities may not make them amenable to visualization by the short interscan times used in OCTA, but it is also possible that their OCT-hyporeflective lumina simply do not provide sufficiently intense reflectivity for time lapse analysis.

## Scleral thickness and choroidal venous outflow

Pang et al. [12] demonstrated congested vortex vein ampullae using UWF ICGA, indicating that CSC eyes have dilated choroidal vessels not just in the macula but throughout the fundus. Choroidal venous drainage exits the eye through the anterior sclera where it is suggested that impaired flow within vortex veins may result in stagnant upstream flow posteriorly where hyperpermeability of the choriocapillaris’ fenestrated endothelium may occur. Eyes with CSC are more hyperopic and have a shorter axial length than normal eyes [40], while CSC rarely occurs in highly myopic eyes, suggesting that ocular dimensions may play a role in the pathophysiology of CSC. Because the vortex veins penetrate the sclera obliquely, with an intrascleral length of ~4 mm, increased outflow resistance due to quantitative or qualitative alterations in the sclera may result in choroidal drainage impairment. To date, most studies evaluating these associations have been in CSC eyes.

Imanaga et al. [16] first measured scleral thickness in CSC eyes using anterior segment OCT 6 mm posterior to the scleral spur, under the 4 recti muscles. Forty-seven eyes of 40 CSC patients and 53 eyes of 47 age- and gender-matched control participants were compared. CSC eyes had significantly thicker sclera than normal controls having similar axial length and spherical equivalent [16]. Similar findings are conveyed in subsequent reports [41–43]. Additionally, Imanaga et al. [44]. used binarized OCT B-scan images of the macula to study how scleral thickness affected choroidal structure. In 111 eyes of 111 CSC patients, the scleral thickness was not correlated with central CT, but it was significantly correlated with choroidal luminal/stromal ratio [44]. This suggests that sclera thickness may play a role in the increase in luminal components of the posterior choroid increases in CSC eyes, resulting in a more pachychoroid-like tomographic appearance. Accordingly, the sclera seems to play a significant role in the development of the pachychoroid disease phenotype. However, while short axial length has been linked to vortex vein asymmetry in CSC eyes, scleral thickening is not significantly correlated with this asymmetry [45]. To ascertain whether the characteristic choroidal structure of CSC eyes is congenital, acquired, or changing over time, longitudinal research is required.

In 2015, using swept-source OCT Spaide and Ryan [46] reported that 64.8% of CSC eyes had loculation of fluid (LOF) in the suprachoroidal space of the posterior pole. In a recent study by Imanaga et al. [47], LOF was found in 62.0% of CSC eyes, and independent factors linked to LOF included thick choroid and thick sclera. Furthermore, ciliochoroidal effusion (CE) observed on anterior segment OCT was present in 19.0% of CSC eyes compared to only 2.0% of normal control eyes [47]. Among all clinical factors investigated, scleral thickening was the sole factor found to be associated with CE development in CSC [47]. This suggests that the mechanism of fluid retention in the choroid in CSC eyes may be due to impaired vortex vein drainage by thickened sclera as well as decreased permeability of transscleral outflow.

In addition to having a serous retinal detachment, CSC eyes also have clinical characteristics that are quite similar to those of uveal effusion syndrome (UES), including short axial length, hyperopia, scleral thickening, choroidal circulatory disturbances, and LOF in the suprachoroidal space [48]. Since microphthalmia is not always present with UES, there may be a significant pathological overlap between CSC and UES. It has been proposed that scleral resection, a standard treatment of UES, may also be effective in severe CSC cases [26]. However, certain features, such as PEDs, focal RPE leaks, gravitational tracks, and fibrin are rarely seen in UES, but may be more specific to CSC. Nonetheless, evidence is accumulating for the involvement of the sclera in the pathogenesis of CSC. Observation of the sclera will be an important target in understanding of the pathogenesis of CSC as well as the other pachychoroid diseases.

## Evolution in the understanding of pathogenesis

**Fig. 2:**
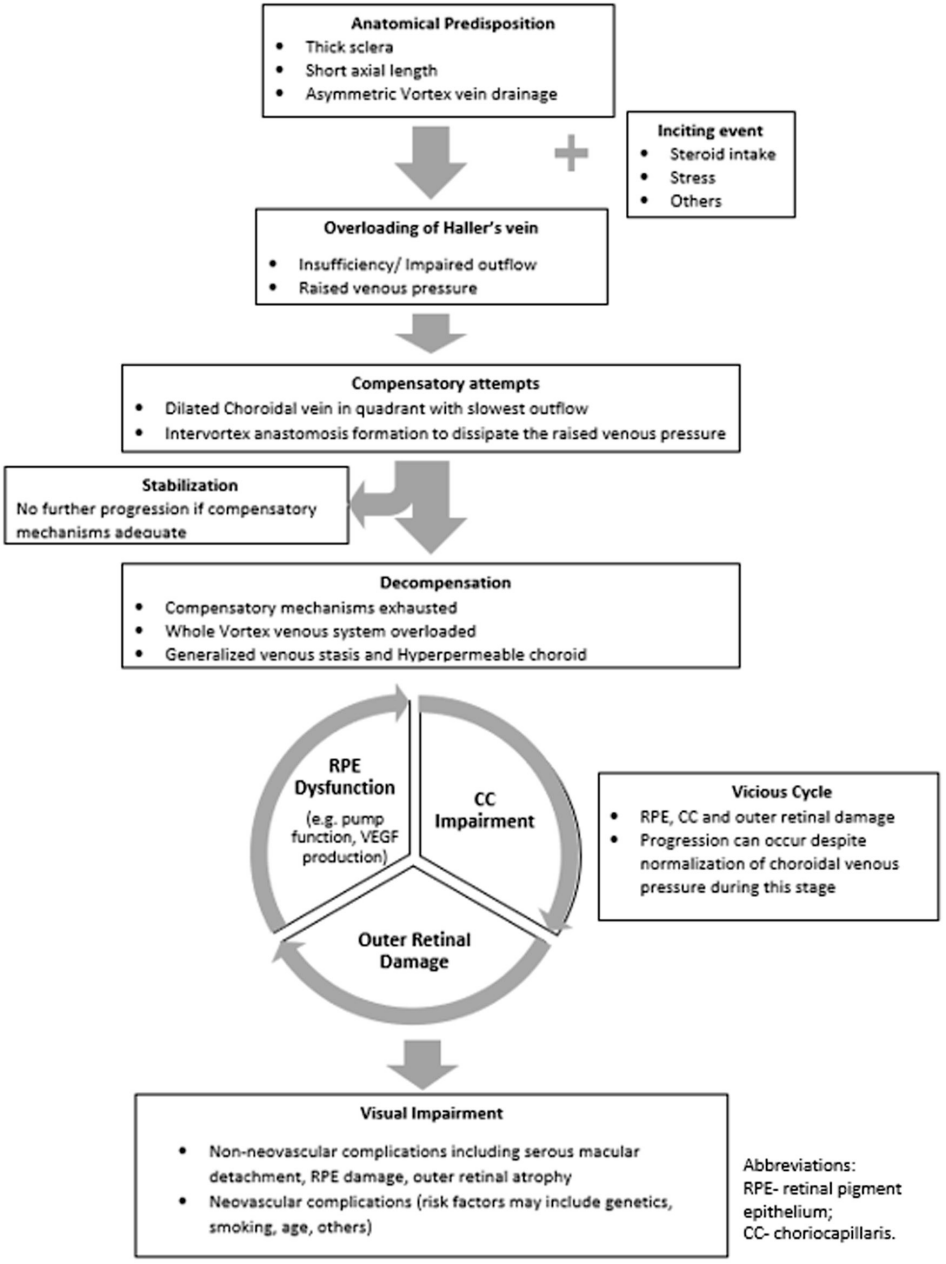
Proposed ‘Multi-hit theory’ in the pathogenesis and evolution of pachychoroid disease.

The mechanisms that underlie CSC and the wider group of the pachychoroid disease spectrum remain somewhat elusive, partly because the hemodynamic and hydrodynamic bases are not fully revealed by histology. Understanding these mechanisms is therefore an exercise in drawing inferences—from ex vivo anatomical findings and from the clues provided by in vivo multimodal imaging—and assembling these into a coherent disease model. While many imaging features have been associated with the pachychoroid disease phenotype, accumulating evidence suggests that the various factors may act in different ways, such as predisposing, inciting, aggravating, and perpetuating. In addition, some features may develop initially as a compensatory mechanism, while others may reflect end organ damage. We propose a “Multi-hit theory” in which the following sequence of events can encompass the imaging findings at the various stages of the pachychoroid disease spectrum: 1) anatomical predisposition; 2) inciting event; 3) compensatory mechanism; 4) saturation, decompensation and vicious cycle, and 5) visual loss. (Fig. 2) Whether an eye progresses through all five stages depends on a complex interplay between the various factors. For example, if the inciting event is short-lived, or if compensatory mechanisms are adequate, venous overloading may resolve.

### Anatomical predisposition

As discussed in “New observations related to choroidal venous alterations”, anatomical risk factors such as short axial length and thick sclera have been associated with CSC. Similarly, asymmetry in the distribution of vortex venous drainage, possibly congenital or developmental in origin, has been implicated in the development of venous congestion. However, these factors likely act as predisposing factors rather than disease-causing factors, as anatomical features remain largely unchanged during an individual’s lifetime. It is therefore likely that other triggers are required to initiate disease. Similarly, the fact that some eyes with pachychoroid disease can resolve spontaneously despite the same anatomical features being present, further points to these features acting as predisposing or aggravating factors.

### Inciting event

Aberrant steroid metabolism and aberrant metabolic response to stress may explain why steroid intake and acute stress may predispose certain individuals to the onset of CSC. Interestingly, abnormal sleep patterns and obstructive sleep apnea have also been found to be major risk factors for CSC. However, these factors do not induce disease in all exposed individuals. Therefore, Hirooka et al. [49]. proposed a two-hit theory in which triggering factors incite disease in individuals with predisposing factors. In support of this “two-hit theory”, it has been reported that, the choroid is significantly thicker in the affected eyes of unilateral CSC patients compared to the contralateral unaffected eyes. However, there is no difference in scleral thickness [50]. This suggests that the scleral thickening may act as a “second hit” in eyes with thickened choroid, contributing towards the development of a serous retinal detachment. Additionally, the sclera is thinner in eyes with steroid-induced CSC than in eyes with idiopathic CSC, indicating a possibly less significant role for the sclera in the pathogenesis of steroid-induced CSC [51].

However, no clear inciting event can be identified in a notable proportion of patients with CSC. The reason behind the higher prevalence of CSC affecting males and the potential role of sex hormones remains unclear [52, 53]. Other proposed associations with hypertension, sleep apnea, infection in CSC also remain unresolved. Depending on the duration and severity of the inciting event(s), the venous overloading effect may be transient or prolonged.

### Compensatory attempts

The development of venous overloading could be considered the first indication of pachychoroid disease disease spectrum. The extent of Haller’s layer congestion and pachyvessels may be restricted to only a single quadrant or may extend to all quadrants. In contrast, a single measurement of subfoveal CT is limited in defining pachychoroid disease because there can be marked regional variability in CT. The thickest areas of the choroid typically occur in the drainage areas of the dominant vortex system where pachyvessels and CVH are found. Hence venous congestion can also be expected to develop following obstruction of the outflow in the affected quadrant(s). Conversely, eyes with hypoplastic vortex venous drainage in 1 quadrant may be less effective at developing adequate compensation to dissipate an increase in venous pressure. If obstruction affects mainly a single ampulla, the increased venous pressure may be dissipated through congestion of the affected quadrant. If this compensatory mechanism is adequate, the condition may stabilize without further progression. If venous pressure remains high despite dilatation of Haller’s veins in the affected quadrant, anastomosis may develop to allow further dissipation of the raised venous pressure to vortex systems in adjacent quadrants. It is also likely that in many eyes with risk factors such as short axial length and thick sclera, more than 1 ampulla may be obstructed at the same time, which will reduce the ability of neighboring quadrants to equalize and dissipate the raised venous pressure in the system.

### Saturation, decompensation, and vicious cycle

If all quadrants have become saturated despite anastomosis formation, the affected eye will exhibit generalized venous stasis and chronic hyperpermeability. A vicious cycle of choriocapillaris ischemia, RPE damage, and outer retinal atrophy ensues, because the ischemic drive can perpetuate the cycle even if the venous congestion eventually abates. This may explain why previous reports have found higher degree of choroidal thickening in CSC eyes compared to that observed in PNV and PCV eyes, in which the choroid is often only moderately thickened. Finally, additional risk factors such as age, genetics, and smoking may explain why only some eyes with pachychoroid disease eventually progress to neovascular complications. While most currently available data support the venous system as the main region of disturbance in the pachychoroid disease phenotype, it is possible that with increasing choriocapillaris loss, artery-to-venous shunting may also contribute to this vicious cycle, as the density of the smaller vessels separating arterial from venous blood diminishes. Brinks et al. have hypothesized that there may even be a role for choroidal arteriovenous anastomosis in the pathogenesis of CSC and other pachychoroid disease entities [54].

Previous researchers have attempted to create an animal model of choroidal congestion by vortex vein ligation [55]. In monkey eyes, vortex vein ligation resulted in choroidal vascular remodelling but not serous retinal detachment [55]. Many features characteristic of pachychoroid disease can be observed, including vortex vein dilation, choriocapillaris filling delay, intervortex venous anastomosis, choroidal thickening, and pulsatile vortex venous flow.

### Visual impairment

Visual impairment in pachychoroid disease is generally caused by a combination of serous macular detachment, RPE damage, outer retinal atrophy, and complications of macular neovascularization. Even in cases with apparently preserved visual acuity, visual function may still be altered in other manners such as metamorphopsia or reduced contrast. In addition to the severity of choroidal congestion, several other factors may also determine the impact on vision. These include the location of maximum congestion, whether the fovea is involved, the duration of pachychoroid disease, and background demographics including age, smoking, and possibly genetic background [53, 56].

## Pachychoroid diseases and management updates

### Update on CSC

Recent studies using multimodal imaging in CSC, particularly UWF OCT and en face OCTA have greatly enhanced our understanding of choroidal venous alterations in CSC [57]. Multimodal imaging enhanced the precision of CSC diagnosis in relation to chronicity and secondary RPE and retinal complications. A new multimodal imaging-based classification system of CSC has recently been proposed in which CSC is classified into one of four categories (simple, complex, atypical or no CSC) [58] (Table 1). This proposed classification system showed moderate agreement (k 0.50 for major criteria; k 0.57 for CSC subgroup classification) among 10 retinal specialists in a cross-sectional study evaluating 61 eyes in 34 patients with presumed CSC [59]. Interestingly, the k value was significantly lower (from 0.6 to 0.2) when the grading was done without prior information about the fellow eye, suggesting that the diagnosis of CSC can be affected by the history of CSC in the fellow eye. Therefore, CSC disease grading should include information from both eyes simultaneously to improve the agreement. Use of this multimodal imaging-based classification showed that eyes with complex CSC were more likely to develop macular neovascularization (MNV) than simple CSC, and MNV development was associated with an older age of presentation. Clearly, further improvement in the proposed classification system is required to achieve higher levels of agreement.

**Table 1:** Proposed Multi-modal imaging-based central serous chorioretinopathy classification by the CSC International Group.

**Simple** Total area of RPE alterations ≤2 DA	**Primary** First known episode of SRF	**±Persistent** SRF > 6 months	**±Outer retinal atrophy** ONL thinning ± ELM disruption ± EZ attenuation	**±CNV**
	**Recurrent** Presence of SRF with history or signs of resolved episode(s)			
	**Resolved** Absence of SRF			
**Complex** Total area of RPE alterations >2 DA or multifocal	**Primary** First known episode of SRF	**±Persistent** SRF > 6 months	**±Outer retinal atrophy** ONL thinning ± ELM disruption ± EZ attenuation **±intraretinal fluid**	
	**Recurrent** Presence of SRF with history or signs of resolved episode(s)			
	**Resolved** Absence of SRF			
**Atypical**	Bullous variant, RPE tear, association with other retinal diseases			

*RPE* retinal pigment epithelium, *DA* disc areas, *SRF* subretinal fluid, *ONL* outer nuclear layer, *ELM* eternal limiting membrane, *EZ* ellipsoid zone, *CNV* choroidal neovascularization.

In addition to choroidal venous alterations discussed in “Evolution in the understanding of pathogenesis”, multimodal imaging also documents changes in CSC consistent with RPE dysfunction and pachydrusen [60]. Pachydrusen have been found to occur in >40% of CSC patients and were associated with an older age [61, 62]. With the use of fundus autofluorescence, it has been demonstrated that CSC eyes with pachydrusen had more extensive RPE abnormalities and that pachydrusen could be localized to 36% of choroidal vascular hyperpermeability sites [62]. Flat irregular RPE detachment on OCT is a useful feature predictive of MNV. Presence of flow signal between RPE and Bruch’s membrane on OCTA is useful to confirm MNV [63].

Based on the currently available evidence, the treatment of choice for symptomatic CSC is half-dose or half-fluence photodynamic therapy (PDT) with verteporfin [64] (Table 2). The efficacy of half-dose PDT has been demonstrated in the multicentre randomized controlled PLACE and the SPECTRA trials [64–66]. The REPLACE and SPECS trials further showed that cross-over to half-dose PDT after previous primary treatment failure following high-density subthreshold micropulse laser treatment (HSML) or oral eplerenone in the PLACE and SPECTRA trials led to improved anatomic and functional outcomes [67, 68]. PDT was shown to be superior over HSML regardless of a focal or diffuse leakage pattern on FA [69]. Functional improvement was demonstrated in an analysis of 57 eyes with chronic CSC treated with half-dose PDT in the PLACE and SPECTRA trials assessed with BCVA and retinal sensitivity [70]. Fundus autofluorescence and OCT imaging also confirmed the safety of half-dose PDT, with no patients developing foveal atrophy. Regarding the optimal timing of treatment, a study showed that patients with longer duration of symptoms were less likely to have visual acuity gain of ≥2 lines after half-dose PDT, with a 4% reduced likelihood for every week of increase in duration of symptoms [71].

**Table 2:** Summary of recent treatment trials in Central Serous Chorioretinopathy.

Study Name	Description of Design	Key findings
PLACE	Randomized chronic CSC patients to half-dose photodynamic therapy (PDT) versus high-density subthreshold micropulse laser (HSML)	Half-dose PDT was superior compared to HSML in patients with chronic CSC in terms of higher rate of resolution of SRF, higher increase in BCVA, greater increase in retinal sensitivity on microperimetry, and larger reduction in the highest macular RPE detachment
SPECTRA	Randomized chronic CSC patients to half-dose PDT versus eplerenone	Half-dose PDT resulted in significantly higher rate of complete SRF resolution compared with eplerenone at 12 weeks (78% versus 17%). At 12 months, BCVA were better in patients who received primary half-dose PDT than those who had delayed PDT with persistent SRF after eplerenone treatment
REPLACE	Cross-over to half-dose PDT after previous primary treatment failure following HMSL	At 1 year, 78% of 32 patients (*p* = 0.030) who crossed over to PDT achieved complete SRF resolution, compared to 67% of 10 patients who crossed over to HSML. The mean retinal sensitivity improved in the PDT group but not in the HSML group.
SPECS	Cross-over to half-dose PDT after previous primary treatment failure following eplerenone	At 3 months after crossover treatment, 87.5% of 37 patients who crossed over to PDT had complete SRF resolution compared to 22.2% of 9 patients who crossed over to HSML. The mean retinal sensitivity improved in the PDT group but not in the HSML group.
VICI	Randomized, double-masked, multicenter placebo-controlled trial which compared the use of eplerenone versus placebo for chronic CSC	After 12 months of treatment, eplerenone was not superior to placebo for improving the BCVA in patients with CSC

*CSC* central serous chorioretinopathy, *SRF* subretinal fluid, *BCVA* best-corrected visual acuity, *RPE* retinal pigment epithelium.

Real-world data from two retrospective studies demonstrated the beneficial effects of half-dose or reduced-fluence PDT for chronic CSC, with both studies showing improved BCVA after treatment [72, 73]. Traditionally, PDT has been guided by both FA and ICGA [64], but recently, a study has demonstrated that FA-guided and ICGA-guided half-dose PDT were both effective in treating non-resolving CSC with significant BCVA improvement and reduction in CMT in both groups [74]. Still, PDT in CSC is often guided based on the abnormalities on ICGA, because choroidal abnormalities on ICGA lie at the basis of CSC, and are often much more extensive than abnormalities in FA. The superiority of PDT in CSC is presumably based on its primary effect on the choroidal abnormalities, which is also reflected in the fact that abnormal choroidal hyperfluorescent changes on ICGA in active CSC decrease to a more normal fluorescence pattern in the treated area after ICGA-guided PDT [75]. The observation that retinal pigment epithelial detachments are often significantly reduced after PDT for chronic CSC also point to the fact that they are secondary to the choroidal abnormalities, and that these can be effectively addressed by PDT [76]. Therefore, ICGA is useful in assessing CSC both for guiding PDT and to diagnose the presence of MNV and PCV [64]. Nonetheless, PDT is not effective in all CSC patients, such as severe, long-standing cases with posterior cystoid retinal degeneration which presumably have irreversible damage to the RPE, choriocapillaris, and larger choroidal vessels that also do not respond to other proposed treatments [77–79].

Systemic therapy with mineralocorticoid receptor antagonists like eplerenone has been proposed as a potential treatment for symptomatic CSC [2]. However, eplerenone has been shown by well-designed randomized controlled trials (VICI, SPECTRA) to be not useful in treating CSC [65, 80] (Table 2).

## Pachychoroid neovasculopathy

In 2012, Fung et al. described a subgroup of patients presenting with type 1 MNV with clinical and imaging findings more consistent with long-standing CSC than with AMD [81]. Subsequently, Pang and Freund introduced the term pachychoroid neovasculopathy (PNV) to describe the condition characterized by type 1 MNV secondary to a pachychoroid disease phenotype, not exclusively secondary to CSC [25]. Characteristic features of PNV include a shallow irregular RPE elevation (SIRE) on OCT, also known as “double layer sign” in the absence of typical AMD signs. The location of type 1 MNV is correlated spatially to areas displaying dilated Haller’s layer vessels and attenuation of inner choroid [82].

With the advent of OCTA, there has been an increasing appreciation of this disease entity. OCTA has been shown to be able to detect neovascularization with high sensitivity while low-grade neovascularization may be difficult to differentiate from choroidal hyperpermeability on ICGA. A study by Demirel et al. reported sensitivity of 97% (OCTA) and 66% (ICGA) in detecting MNV in a study evaluating eyes with pachychoroid [83]. The diagnosis of PNV is confirmed when a tangled vascular network corresponding to the SIRE is detected on OCTA. The type 1 MNV of pachychoroid disease are often well visualized by en face OCTA, perhaps more consistently than those of neovascular AMD. This may be due to i) their gradual and insidious onset which allows the vascular networks to become mature by the time it is detected; ii) their relatively planar geometry; and iii) absence of drusenoid material which might otherwise mask the OCT signal. It has been shown that a large proportion of SIRE, seen on OCT cross-sections in eyes with pachychoroid disease, is seen to harbor type 1 MNV when imaged with OCTA and/or dye-based angiography, making the SIRE a sensitive structural OCT biomarker for neovascularization in this context [84]. However, there is no definite consensus regarding the nomenclature, definition, classification, and diagnostic criteria of neovascular pachychoroid disease. For example, PNV and PCV/aneurysmal type 1 (AT1) have been reported as separate entities in many studies; however, others have included both types (with or without aneurysmal lesions) within the PNV category. This inconsistency makes it challenging to compare findings across different studies. In this review, we shall specifically use the term PNV to refer to the entity without aneurysmal lesions.

Data on the incidence and prevalence of PNV are limited. In a recent Japanese study, among the cases with MNV secondary to AMD, the estimated prevalence of PNV and MNV with aneurysmal lesions were about 25% and 40%, respectively [85]. PNV is frequently detected during non-exudative phase. About 20% of fellow eyes of patients with PCV/AT1 have non-exudative MNV. Eyes with non-exudative PNV should be carefully monitored since these lesions carry a high risk of exudative conversion [86]. A study reported that exudative changes occurred in 9% (5/55) of eyes with non-exudative PNV during ~12 months of longitudinal observation [87]. Non-exudative PNV may progress to develop SRF, and aneurysmal lesions may develop from PNV. Occasionally, a large RPE detachment may develop at the terminal of a PNV, which is associated with aneurysmal lesions or abnormal vascular nets [88]. PNV and PCV/AT1 have been suggested to represent a disease continuum. Siedlecki et al. observed that 5 of 37 (13.5%) eyes developed PCV/AT1 from PNV after a mean of 3.4 years [89].

**Fig. 3:**
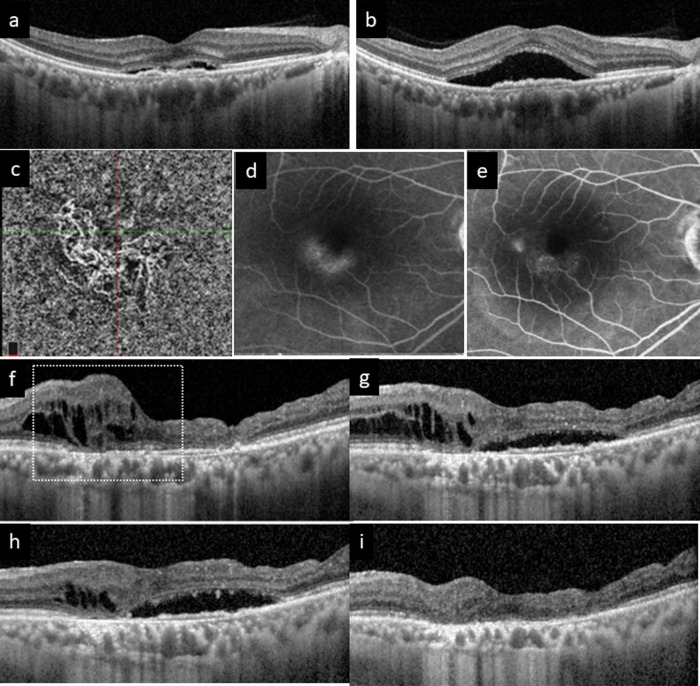
Examples of the pachychoroid disease spectrum. **a**–**e** A case of pachychoroid neovasculopathy (PNV) complicating central serous chorioretinopathy (CSC). **a** OCT at baseline and (**b**) OCT at follow-up demonstrated worsening of subretinal fluid over a shallow irregular retinal pigment epithelial elevation. Thickened choroid with pachyvessels can be observed at both visits. **c** OCTA at baseline detected the presence of vascular flow signal due to macular neovascularization. Fluorescein angiography (FA) was performed at baseline (**d**) and repeated one year later (**e**). Note the leakage pattern was different at the two visits: an occult leakage pattern in keeping with type 1 MNV was seen at baseline (**d**). In contrast, smoke-stack pattern was seen arising from a focal point in (**e**), suggesting CSC-related mechanism was likely the drive for subretinal fluid at this timepoint. **f**–**i** Another case of PNV with varying amount of intraretinal fluid (IRF) and subretinal fluid (SRF) at different visits. Severe atrophy of RPE and outer retinal layers including the external limiting membrane are observed. During follow-up, the amount of IRF and SRF fluctuates. It is postulated that the IRF originating from the choroid and/or macular neovascularization enters the neurosensory retina in areas with both RPE and outer retina (external limiting membrane) defects. In addition, choroidal congestion and exudative MNV may contribute to SRF in areas where the outer retina remains intact (**g**, **h**). The fluid in both compartments disappeared after a single injection of brolucizumab (**i**). Note also the reduction of choroidal thickness after treatment in (i) compared with (**f**, **g**), which may play a role in fluid resolution.

Regarding treatment, eyes with PNV seem to have lower requirement for anti-VEGF injections compared to typical neovascular AMD (nAMD) [90]. The level of aqueous VEGF has been reported to be lower in eyes with PNV than in nAMD [91]. In particular, aflibercept appears more effective in fluid absorption than ranibizumab, which may be attributable to the greater effect on the choroid manifested by the decrease of choroidal vascular hyperpermeability and reduction of choroidal thickness [92]. However, some eyes with PNV are refractory to intravitreal anti-VEGF monotherapy and “rescue PDT” appears useful in these cases [93]. Some investigators have reported that initial combination therapy using PDT and anti-VEGF is effective and well-tolerated with the benefit of reducing the burden of anti-VEGF injection in the maintenance phase [94, 95]. In some cases, despite multimodal imaging, it is difficult to clearly discern whether MNV or CSC is the cause of SRF and it is likely that there is a mixture of both factors [96]. Combination therapy may be a treatment option in these cases. In the setting of pachychoroid disease, SRF could originate from CSC-related mechanism and might not always be a sign of MNV activity. FA leakage pattern can aid in discerning the origin of SRF (CSC versus exudative or non-exudative PNV, Fig. 3) [63, 97]. Also, choroidal fluid may enter into the retina causing intraretinal cysts when there are severe defects in the RPE and outer retina, including the external limiting membrane (Fig. 3) [98].

## Polypoidal choroidal vasculopathy/aneurysmal type 1 neovascularization

Idiopathic PCV was first described by Yannuzzi et al. [99]. Although the initial description of this disease considered PCV to be a disease of the inner choroid, newer multimodal imaging has demonstrated that PCV/AT1 is in fact a variant of type 1 (sub-RPE) neovascularization [100]. In 2018, Dansingani et al. proposed the term AT1 as a generic descriptor to describe a variant of type 1 MNV with aneurysmal (polypoidal) lesions [101]. The term reflects the idea that the lesion is neovascular in nature and primarily vascular (an aneurysmal dilatation) rather than epithelial (a fleshy or solid polyp). The authors suggested that AT1 might be used as a descriptive term that is not disease-specific. The presence of aneurysmal lesions has also been recognized in neovascularization associated not only with pachychoroid disease but also with typical drusen-associated nAMD, myopic staphyloma, radiation retinopathy, and angioid streaks [102]. Although aneurysmal lesions are generally less common in MNV with a predominantly drusen-driven background, they may develop at the terminal of chronic type 1 MNVs. Subsequently, the term “aneurysmal PNV” has been proposed to reflect the subset of AT1 associated with pachychoroid phenotype, in which aneurysmal lesions are hypothesized to evolve from PNV. In this review, we will keep the term PCV/AT1 for this entity.

The Asia-Pacific Ocular Imaging Society (APOIS) PCV workgroup recently recommended the terms polypoidal lesion and branching neovascular network for the two key lesion components in PCV/AT1 [103]. The term “polyp” is inappropriate because strong evidence based on OCT and histologic analysis suggests these lesions are not fleshy, solid. The term “aneurysmal” represents the dilated vascular structure: however, internal vascular structures may be seen within some lesions. Although the panel recognized the potential benefit of the terms “aneurysm” and “aneurysmal”, they recommended the term “polypoidal lesion” until clearer and more universally accepted evidence is available. They also recognized that branching vascular network and PNV may represent a continuum and recommend the term branching neovascular network to better reflect the neovascular nature.

It is believed that most PCV/AT1 lesions in the Asian population is driven by a pachychoroid-related mechanism. A previous study conducted in Korea including >300 eyes reported a mean subfoveal CT of ~270 μm (range 40–650 μm). In ~90% of patients, irrespective of CT, pachyvessels were observed below the presumed origin of the branching vascular networks feeding the aneurysms associated with overlying attenuation of the inner choroidal layers, which aligns with the pachychoroid disease definition [8, 104]. Recent studies, encompassing Asian and Caucasian eyes with PCV/AT1, support this hypothesis, while also suggesting relatively fewer Caucasian patients with a pachychoroid-related mechanism. In a study comparing 128 Asian and 122 Caucasian patients, more Asian eyes had pachyvessels (84.4% vs 28.7%) and choroidal vascular hyperpermeability (70.3% vs 17.2%) [105]. This finding was consistent with another study which revealed that Caucasian patients had a significantly higher prevalence of soft drusen (50.0% vs 25.0%), and a significantly lower prevalence of pachyvessels (41.3% vs 62.5%) [106]. However, in several Japanese studies, ~40% of cases with aneurysmal lesions were also classified into the nAMD category [107]. This discrepancy may be attributable to the ambiguity of the pachychoroid disease definition (e.g. presence of pachychoroid disease features below the MNV origin sites) as well as the challenge in differentiating the various drusen subtypes (e.g. pachydrusen vs. non-confluent soft drusen). There has been a great variability in the prevalence of soft drusen in eyes with PCV/AT1 or in the fellow eyes among Asian population, ranging from 4% to 50% [105, 106, 108]. Also, the boundary between pachychoroid-, and drusen-driven mechanism remains ambiguous (e.g. cases displaying both the presence of a few intermediate or large soft drusen and a phenotype of pachychoroid without apparent thickening).

Drusen are a hallmark of AMD and many studies have demonstrated a lower prevalence of drusen in eyes with PCV/AT1 compared to eyes with typical nAMD. However, pachydrusen have been found in up to 60% of eyes with PCV/AT1 [62, 108]. Pachydrusen differ from conventional soft drusen in their shape and distribution, and are associated with increased Haller’s layer thickness and attenuation of the choriocapillaris [61, 109, 110]. Certain pachydrusen, particularly the isolated type, is similar to soft drusen in appearance [111]. However, whether pachydrusen shares the genetic background with soft drusen and confers similar risk for MNV remains unclear. In a retrospective cohort study including 632 eyes with intermediate AMD, the 5-year cumulative incidence of progression to nAMD was 17.8% in eyes with soft drusen and 17.0% in eyes with pachydrusen. In reference to soft drusen, pachydrusen were associated with progression to PCV/AT1 and not to typical nAMD [112]. In another study exploring the association between pachydrusen and PCV/AT1, the location of exudation was not co-localized with pachydrusen, and there was no increase in pachydrusen size over time [113]. Large-scale studies including fellow eyes of unilateral PCV/AT1 revealed that the 5-year incidence of exudative MNV was approximately 10%. There was no correlation between pachydrusen and the occurrence of exudative MNV. The authors postulated that presence of pachydrusen could merely represent an epiphenomenon of the pachychoroid disease spectrum [114]. Recently, age-related scattered hypofluorescent spots on late-phase ICGA (ASHS-LIA), with no corresponding abnormalities on other imaging modalities, were proposed as a possible precursor of PCV/AT1. These hypofluorescent spots were seen in about 60% of 187 patients with PCV/AT1. The authors speculated that ASHS-LIA represents neutral lipid accumulation in all its forms, i.e., within Bruch’s membrane, pre-basal linear deposits, and basal linear deposits, thus representing the total burden of lipoprotein accumulation over adulthood that culminates in soft drusen in AMD. ICG dye normally passes through Bruch’s membrane and is taken up by the RPE, resulting in a homogeneous background fluorescence in late-phase ICGA. It was proposed that these accumulated lipids may impede ICG dye’s access to the RPE, resulting in hypofluorescent spots in late-phase ICGA [115]. Alterations in the biomechanical properties of Bruch’s membrane as a result of these basal laminar deposits may contribute towards the propensity of PNV and PCV/AT1 lesions to remain below the RPE, and/or a higher occurrence of hemorrhage in PCV/AT1.

For diagnosing PCV/AT1 and differentiating it from typical nAMD, ICGA remains the best validated method. However, the use of ICGA is expensive, invasive, and not always available at clinical centers. Several studies have shown potential diagnostic features on OCT and fundus photography for detecting PCV/AT1 without the use of ICGA [116–118]. The APOIS PCV workgroup developed and reported a set of non-ICGA-based imaging criteria that can differentiate eyes with PCV/AT1 from typical nAMD. The combination of 3 OCT-based major criteria (sub-RPE ring-like lesion, complex RPE outline on en face OCT, and a sharp-peaked RPE detachment) achieved an AUC of 0.90. Four additional features (orange nodule, thick choroid with dilated Haller’s layer, complex or multilobular RPE detachment, and double layer sign) met the prespecified AUC requirement for minor criteria [103].

As mentioned in “Pachychoroid neovasculopathy”, OCTA can delineate type 1 MNV in PNV. Similarly, OCTA can also reveal the branching neovascular network within the PCVAT1 complex. However, the appearance of the aneurysmal/polypoidal lesions is variable. Some studies have reported these lesions to show increased flow signal, while others have reported reduced flow signal. These variations may relate to the use of different OCTA instruments in acquiring the data, and different segmentation strategies in analysis. There remains ongoing controversy over the vascular structure of polypoidal lesions. Bo et al. found that polypoidal lesions (all 43 lesions from 23 eyes) consisted of densely or loosely tangled vascular structures at the margins of branching vascular networks, and proposed that many polypoidal lesions lack an aneurysmal structure [119]. The same group documented the evolution of five eyes in which the polypoidal lesion evolved into a typical type 1 MNV after multiple anti-VEGF injections and argued that this further supports that the initial lesion was not an aneurysmal lesion. Recently, using 3-D reconstruction of swept-source OCTA, Teo et al. demonstrated all polypoidal lesions exhibit internal vascular architecture in the form of coil-like loops, while small focal nodules were detected in 70%. These OCTA findings are consistent with prior observations by Yuzawa et al. in 2005 using high-speed ICGA. They described several patterns of internal architecture of polypoidal lesions, including small clusters of aneurysms, larger aneurysmal dilatations, and large vessel deformations such as loop or coil-like configurations, constrictions, and dilatations [120]. A factor contributing to the confusion in the literature is the lack of standardization arising from the term “polyp” or “polypoidal lesion”. Some researchers use this term to refer to the whole vascularized RPE detachment within which focal vascular dilatations can be found. However, with confocal scanning laser ophthalmoscope-based ICGA performed simultaneously with OCT and/or OCTA, it may be easier to interpret the complex internal architecture within the vascularized RPE than when using flash camera-based (flood-illuminated) ICGA.

When treating PCV/AT1, the aneurysmal lesions were previously the main target, as they were recognized as the most likely source of hemorrhage and exudation. Closure of the aneurysmal lesions with thermal laser or subsequently with PDT has been widely used [121]. With cumulating favorable clinical experience of intravitreal anti-VEGF monotherapy, the primary treatment of choice has shifted from PDT to anti-VEGF injections [122]. Concern regarding to the potential damage to choroidal vasculature and RPE associated with repeated PDT, especially when using full-fluence settings, has also led to a reduced use of PDT [123]. Thermal laser photocoagulation can still be a useful option to treat focal PCV/AT1 lesions outside the central macula, especially when PDT and/or anti-VEGF therapy are not available [124].

Currently, intravitreal anti-VEGF therapy monotherapy or combined with PDT is commonly used as the standard treatment of symptomatic macular PCV/AT1 [100]. As described in our previous review, the EVEREST II and PLANET studies continue to provide level 1 evidence for these treatment options (Table 3) [125–128]. Head-to-head comparison between two studies may be unreliable due to the differences in study design and possibly different study populations. Nevertheless, these results may suggest differences in the treatment effects of two drugs, favouring aflibercept for PCV/AT1. Due to the low event rate of rescue treatment in the PLANET study, limited conclusion could be drawn regarding the role for rescue PDT. Many investigators have searched for biomarkers related to choroidal morphology, which can guide the optimal treatment, but findings have been inconsistent [123, 127, 129, 130]. Recognizing that all components of the lesion are neovascular in nature, the anti-VEGF treatment regimens in PCV/AT1 have also progressively become more aligned with those used to treat typical nAMD. In addition, given the great inter- and intra-individual variability in injection frequency required as well as the tendency for acute hemorrhage, proactive and individualized treat-and-extend (T&E) regimens have gained acceptance among retinal specialists [130, 131]. Several studies have demonstrated that intravitreal aflibercept monotherapy, following a T&E protocol with a 4-weekly increment and a maximum interval of 12- or 16- week is an acceptable option [132].

**Table 3:** Summary of key treatment trials for polypoidal choroidal vasculopathy.

Study Name	Description of Design	Key findings
EVEREST II	Randomized controlled trial comparing combination therapy with ranibizumab (IVR) and PDT vs IVR monotherapy	combination therapy with ranibizumab (IVR) and PDT achieved. • Superior visual gains (8.3 vs 5.1 letters at month 12, and 9.7 vs 5.6 letters at month 24) • Higher rate of polypoidal lesion regression (69.3% vs 34.7% at month 12, and 56.6% vs 26.7% at month 24) compared to IVR monotherapy arm. • Proportion of participants with disease activity was lower in the combination group (27.0% vs. 54.3%) at month 24. • Initial combination therapy has the advantage of reducing the need for retreatment with ranibizumab over 24 months
PLANET	Randomized controlled trial comparing intravitreal aflibercept (IVA) monotherapy vs IVA with rescue PDT	• IVA monotherapy group was non-inferior to the IVA + rescue PDT group: • BCVA gain achieved at week 52 was (+10.7 letters) and (+10.8) in the monotherapy and IVA + rescue groups, respectively • The vast majority of patients in this trial were treated with IVA monotherapy as only 17.0% met the rescue criteria over 96 weeks • Among the small subgroup who met the criteria for rescue PDT, continuing an IVA monotherapy was non-inferior to active rescue PDT (+2.6 vs 0 letters). Most eyes (nearly 80%) had a fluid-free retina at the end of the study, despite about 30% of eyes achieving polypoidal lesion regression on ICGA • the addition of PDT to patients who did not respond initially to IVA monotherapy did not provide any benefit
HAWK Japanese subgroup	Randomized controlled trial comparing intravitreal brolucizumab up to 12 weekly vs IVA	• brolucizumab q12w/q8w monotherapy resulted in robust and consistent BCVA gains that were comparable to q8w aflibercept dosing. • Anatomical outcomes favored brolucizumab over aflibercept, with 76% of brolucizumab participants maintained on q12w dosing over 2-year
TENAYA & LUCERNE	Randomized controlled trial comparing intravitreal faricimab up to 16 weekly vs IVA	• PCV subgroup results not available due to small number of PCV patients
PULSAR PCV subgroup	Randomized controlled trial to evaluate the safety and efficacy of 8 mg q12w or IVA 8 mg q16w compared to IVA 2 mg q8w after 3 loading injections	• increases in BCVA (~9 letters) from baseline at week 48 were observed in all groups. • about 85% of patients receiving IVA 8 mg were maintained on treatment intervals ≥12 weeks. • The safety profile of IVA 8 mg was similar compared to the known safety profile of 2 mg.

Brolucizumab is a novel single-chain fragment variable antibody that inhibits all isoforms of VEGF-A. In phase 3 HAWK and HARRIER trials, brolucizumab was found to be similarly efficacious but more durable than aflibercept for the treatment of nAMD [133]. Its low molecular weight may allow the delivery of higher drug concentration, potentially more effective tissue penetration, and increased duration of action. In the subgroup analysis of Japanese patients with PCV/AT1 from the HAWK trial, brolucizumab q12w/q8w monotherapy results were comparable to aflibercept q8w dosing [134]. Other short-term studies also revealed that brolucizumab led to robust drying with a possibility of an extended treatment interval in the treatment naïve and “switched” cases. Aneurysmal lesions regressed in 80–90% after three loading injections, and RPE detachments refractory to other drugs revealed significant anatomical improvements after a single injection [135–137]. Subfoveal CT significantly decreased by 15–20% after 3 monthly injections, which correlated with resolution of retinal fluids. These positive results might be attributable to the better penetration of the drug through retinal layers. It has been suggested that brolucizumab may cause significant anatomic changes in the choroid possibly exceeding those previously reported for other anti-VEGF agents, which is beneficial for the treatment of MNV driven by the pachychoroid phenotype [138]. Despite these promising findings, safety issues with regards to intraocular inflammation (IOI) ranging from iritis to retinal vasculitis with or without vascular occlusion remain a major concern. In Asian patients with PCV/AT1, IOI was noticed in about 9–19%, most of which were controlled well with corticosteroids (topical, subtenon, intravitreal, or systemic). However, occlusive vasculitis resulting in moderate to severe visual loss occurred in about 1–2% of cases [137]. The PROUD study is now being conducted to investigate the efficacy and safety of brolucizumab in patients with PCV/AT1 in Korea.

Faricimab is a bi-specific antibody recently approved for nAMD and diabetic macular edema targeting angiopoietin-2 and VEGF-A simultaneously. Angiopoietin-2 is a ligand that plays a key role in vascular destabilization and inflammation. This dual mechanism has the potential to provide sustained efficacy through durability of effect beyond anti-VEGF therapy alone in nAMD. The 2-year TENAYA/LUCERNE data revealed vision stability and tight fluid control with faricimab up to q16w dosing, with fewer injections compared to aflibercept. These visual and anatomic outcomes were achieved with approximately 80% of faricimab-treated patients on 12-week or 16-week dosing intervals, with an acceptable safety profile [139]. These results were repeated in the subgroup analysis of patients from Asian countries [140]. However, the number of patients with confirmed PCV/AT1 was too small due to the optional nature of ICGA in these trials. A recent report by Mukai et al. on the 3-month outcomes of patients (22 eyes) treated with 3 loading injections of faricimab showed complete polyp regression in 50% and dry macula in 82% [141]. The SALWEEN study (ISRCTN69073386) is being conducted to investigate the efficacy, safety and durability of faricimab in patients with PCV/AT1 in Asia.

A novel intravitreal formulation of Aflibercept delivers 8 mg in 70 μL, providing a 4-fold higher molar dose compared with aflibercept 2 mg. This formulation is hypothesized to provide longer effective vitreal concentrations and enable a more sustained effect on VEGF signaling. The PULSAR trial is being conducted to test the efficacy and safety of IVA 8 mg in patients with nAMD, and recently 96-week results demonstrated similar efficacy, durability, and safety profile in the PCV/AT1 subgroup compared to the overall study population.

## Treatment of other pachychoroid disease variants

Other conditions which reside within the pachychoroid disease spectrum include PPE, PPS, and the more recently described pachyvitelliform maculopathy (PVM). Although focal choroidal excavation (FCE) was previously included, recent evidence indicates that FCE may be a finding resulting from inflammation involving the outer retina, RPE and choroid producing defects in Bruchs membrane and focal choroidal thinning. The presence of pachychoroid disease may simply be a predisposing risk factor for FCE formation in eyes developing these lesions. As already covered in our previous review, the other pachychoroid disease entities represent a spectrum with shared choroidal alterations. Eyes have been noted to evolve from one condition to another over time (e.g. from PPE to CSC) [2]. Yagi et al. followed up 148 eyes with PPE in patients presenting with unilateral CSC and reported that 16.7% of patients progressed to CSC over a follow-up period of 6 years. CVH and increased subfoveal CT were factors associated with progression [142]. PPS is characterized by thickening of the nasal choroid and peripapillary fluid pockets. The optimal treatment for the intraretinal fluid remains unclear. Some authors have reported success with PDT in a pilot series of 25 eyes [143], and favorable response to intravitreal aflibercept has been reported in 2 eyes [144]. However, spontaneous resolution might occur [145], and the overall visual outcome was relatively favorable, associated with decreased intraretinal fluid and decreased choroidal thickening in a long-term follow up study [146]. Treatments may be considered in selected patients, especially those with subfoveal SRF. A properly designed study with masking and a comparator group would be necessary to determine the efficacy of any treatment modality.

PVM is characterized by the accumulation of acquired vitelliform materials overlying pachyvessels. PVM is associated with RPE disruption on OCT and 41% of patients have been reported to develop macular atrophy [147]. Differentiating PVM from geographic atrophy is important as the progression rate, pattern, and response to complement inhibitor therapy are likely different.

## Limitations

This review aims to focus on summarizing learnings from clinical observations and recent treatment trials related to pachychoroid disease spectrum. Based on the cumulating new observations, the authors theorize and propose the multi-hit theory which may encompass the imaging findings at different stages of disease. Further studies, particularly longitudinal data will be required to validate this proposed theory.

Other areas of important progress which have not been included in detail in this review include studies in basic science, genetics associations, animal models and artificial intelligence.

## Future research and conclusion

1. Clarification of definition and nomenclature around “pachychoroid” concept, supported by longitudinal studies to understand evolution.
2. Leverage on increasingly powerful imaging tools for deep phenotyping, focus on choroid and polypoidal/aneurysmal lesions.
3. International collaborations to understand the influence of ethnicity, genetics, and epigenetics.
4. Develop choroid-guided treatment strategies.

In conclusion, this review has summarized recent findings on clinical observations and hypotheses related to pathogenesis of the pachychoroid disease spectrum. We have expanded the discussions from our previous review to cover considerations on the definition, pathogenesis, and limitations in current understanding. The diagnosis of pachychoroid disease spectrum should only be made after adequately excluding choroidal inflammatory or infiltrative diseases. We recognize the limitation related to the prefix pachy-, and associated terms including pachychoroid, pachyvessels, and pachydrusen. However, proposing changes to the current terminology may pose problems as these terms have already been widely used in the literature. As highlighted in this review, current hypotheses do not explain all the observations and heterogeneity observed. As new evidence cumulates and our understanding continues to evolve, any new terms proposed now may also become inadequate. Nevertheless, we encourage readers to not take the “pachy” prefix literally and recognize that this phenotype is not defined by CT alone. Overall, the body of research in this topic has led to the discovery of a unifying phenotype that can potentially explain several common clinical entities and generate new ideas for therapy which address the underlying pathogenic mechanism.

## Methods

This review provides an update to the previously published review on the pachychoroid disease spectrum by this group in 2018. The current review focuses on new data based on a search of peer-reviewed published papers relevant to the pachychoroid disease spectrum from January 2019 onwards, available on the PubMed database (Appendix 1). In order to cover the latest developments, this review will not repeat discussing concepts that have already been covered in our previous review in this journal in 2019. While we aim to avoid repetition of concepts already covered in our previous review, fundamental and important relevant literature published before 2018 is also referenced.

## Supplementary information

Appendix 1
