# Supplementary material for: Pachychoroid disease: review and update
Source: Eye (Lond). 2024 Aug 3;39(5):819–34. doi: 10.1038/s41433-024-03253-4 (PMC11933466; doi:10.1038/s41433-024-03253-4)
Supplement: Supplementary file 1 — Appendix 1 [file 41433_2024_3253_MOESM1_ESM.docx]

Appendix 1. Search strategy

We have performed the following literature searches based on publications between January 2019 and December 2023, in English language:

1. ‘Pachychoroid Disease’
2. ‘pachychoroid’ AND ‘vortex vein’
3. ‘pachychoroid’ AND ‘sclera’.
4. ‘Central serous chorioretinopathy’ AND ‘treatment’ (limited to randomized trials)
5. ‘Polypoidal choroidal vasculopathy’ AND ‘treatment’ (limited to randomized trials)

Articles were screened by GC to remove publications which do not contain relevant or new information related to the topic. Where multiple publications report similar findings, studies and reviews with confirmatory findings will not be included.
